# Adsorption Mechanism of Nitrogen in CNT-Reinforced Silica Aerogels: A Molecular Dynamics Insight

**DOI:** 10.3390/gels12050371

**Published:** 2026-04-28

**Authors:** Wenping Yue, Yiming Song, Jingjing He, Yi Yang, Kaiqi Wei, Yuxuan Liu, Jia Bai

**Affiliations:** 1 Shaanxi Key Laboratory of Safety and Durability of Concrete Structures, Mountains and Rivers Institute of Engineering Science, Xijing University, Xi’an 710123, China; 2 School of Resources Engineering, Xi’an University of Architecture and Technology, No. 13 Yanta Road, Xi’an 710055, China; 3 Power China Northwest Engineering Corporation Ltd., Xi’an 710065, China; 4 College of Military Basic Education, Engineering University of PAP, Xi’an 710086, China

**Keywords:** silica aerogel, carbon nanotubes, molecular dynamics simulation, nitrogen adsorption, porous composites

## Abstract

Silica aerogels are ideal candidates for gas adsorption due to their exceptional porosity and high specific surface area; however, the inherent mechanical fragility of their skeletal framework significantly compromises their operational stability in engineering applications. While the incorporation of carbon nanomaterials effectively enhances the mechanical robustness of aerogels, the specific microscopic mechanisms by which filler microstructure and surface chemistry dictate gas adsorption behavior remain insufficiently understood. In this study, we employed all-atom molecular dynamics (MD) simulations to develop a model of silicon-based porous composites synergistically doped with carbon nanotubes (CNTs) and graphene. The adsorption and diffusion characteristics of nitrogen (N_2_) were systematically investigated across a CNT doping concentration range of 5% to 20%, and the influence of surface hydrophilicity/hydrophobicity on adsorption performance was quantitatively analyzed by modulating potential energy parameters. Our results demonstrate that the introduction of CNTs reconfigures the porous architecture, leading to an approximately 18.25% increase in the normalized specific surface area, which subsequently drives a 15% enhancement in the overall adsorption capacity of the composite. Nevertheless, analysis reveals that the weight-specific adsorption efficiency of the CNT component itself exhibits a declining trend as the doping concentration increases. This phenomenon is primarily attributed to the convex curvature of the CNTs, which restricts the effective contact area and weakens the adsorption potential, alongside the steric hindrance effects arising from local filler agglomeration at higher concentrations. Furthermore, surface chemical properties exert a significant regulatory influence on adsorption; a strongly hydrophilic modified surface (λ = 1.5) achieved an adsorption capacity approximately 98% higher than the baseline condition—an improvement that exceeds the gains provided by purely physical volume expansion. This research elucidates the synergistic mechanism between physical architecture and surface chemical modification in the adsorption process, suggesting that while the physical architecture determines the abundance of potential adsorption sites, the surface chemistry governs the actual efficiency of site utilization. These findings provide critical theoretical insights for the future design of composite aerogel materials that balance structural stability with superior adsorption performance.

## 1. Introduction

Driven by the rapid iteration of clean energy technologies and the increasingly stringent standards for environmental governance, the development of advanced gas adsorption materials has expanded into emerging frontier fields, such as hydrogen energy utilization [1] and trace pollutant capture [2]. As a pivotal technology for achieving high-density physical storage of energy carriers (e.g., hydrogen and methane) and deep separation within complex multi-component systems, adsorption processes based on porous solids have demonstrated immense potential in enhancing energy conversion efficiency and reducing process energy consumption [3]. Compared with traditional separation methods, modern adsorption techniques enable precise molecular recognition and highly efficient enrichment under relatively mild conditions [4]. However, these burgeoning application scenarios often entail extreme operational requirements, such as structural stability under ultra-high pressures or mass transfer durability during rapid pressure-swing cycles [5,6]. Consequently, designing and constructing novel porous media that possess both ultra-high adsorption capacity and exceptional mechanical robustness is not only a prerequisite for overcoming existing engineering bottlenecks but also a vital direction for continuous exploration in the field of materials chemical engineering [7].

Among various porous adsorption media, silica aerogels have demonstrated significant potential for gas capture, owing to their exceptionally high porosity and extensive mesoporous network structures [8]. However, the silica framework, which is composed of randomly aggregated nanoparticles, suffers from inherent mechanical brittleness. This structural deficiency often leads to microstructural shrinkage or even densification when the material is subjected to external pressure fluctuations or capillary forces [9,10]. To address this mechanical shortcoming, Moner-Girona et al. [11] systematically investigated the micromechanical response of aerogels using microindentation techniques; their findings indicated that the skeleton of pure silica aerogels is extremely fragile and can hardly withstand even minor shear stresses, which severely compromises its structural stability. Furthermore, research by Soleimani Dorcheh and Abbasi [12] confirmed that during medium removal or desorption processes, the peak capillary pressure generated within the pores can reach as high as 200 MPa. Such immense internal stress is the primary cause of irreversible framework collapse and the subsequent loss of porosity. This structural instability may result in a reduction of the effective pore volume, thereby limiting the reliability of the material in operating conditions that require frequent adsorption–desorption cycles [13,14]. Recent investigations into microstructural evolution have further highlighted that understanding the damage characteristics and structural stability of such porous frameworks is essential for long-term performance [15].

To rectify these mechanical deficiencies, a prevalent modification strategy involves incorporating carbon nanotubes or graphene, materials characterized by their high elastic moduli, into the aerogel matrix as reinforcing phases [16,17]. Such molecular-level design and reconstruction strategies are increasingly recognized for their ability to tailor material properties at the nanoscale [18]. Previous studies have empirically validated the efficacy of carbon nanomaterials in enhancing the Young’s modulus and compressive strength of aerogels at the macroscopic level. For instance, experimental research by Meador et al. [19] demonstrated that the integration of carbon nanofibers into the silica aerogel network significantly augments the overall mechanical performance with negligible increases in material density. Similarly, reinforcing composite systems with distinct phases has proven effective in achieving superior mechanical strength and structural integrity [20]. Furthermore, Dervin et al. [21] investigated graphene oxide (GO) doped systems and found that a trace addition of only 0.5 wt% GO reduced the volumetric shrinkage during the drying process by 19%, effectively bolstering the porous framework’s resistance to collapse. Recent advancements have further highlighted that the scalable synthesis and unique surface characteristics of graphene oxide, particularly those derived from sustainable alternatives like biochar, are crucial for tailoring the mechanical and adsorptive properties of such composite frameworks [22]. A finding supported by recent work on how carbon-based nanomaterials effectively modulate the structural and thermal properties of composite frameworks [23]. Within the design paradigm of such composite systems, it is generally conceptualized that distinct components possess specific functional priorities: the silica matrix, with its extensive micropore distribution, primarily serves to provide adsorption sites for the selective sequestration of target gas molecules [24]; meanwhile, the rigid carbon nanostructures predominantly function as mechanical scaffolds, aimed at preserving the integrity of the pore structure by enhancing the overall skeletal stiffness to resist deformation, thereby effectively preserving the adsorption performance of the material [25].

Although the macroscopic efficacy of carbon nanomaterials in reinforcing the mechanical properties of aerogels has been extensively validated [26], a profound understanding of how rigid fillers specifically influence the gas adsorption behavior of porous systems at the microscopic scale remains elusive. On one hand, the introduction of fillers inevitably reconfigures the pore network of the material; their geometric configurations and dispersion states not only dictate the structural support efficiency of the framework but may also alter the local residence environment of gas molecules through steric hindrance or curvature disparities [27,28]. Theoretical calculations on individual carbon materials have suggested the existence of such geometric effects: quantum chemical studies by Umadevi and Sastry [29] indicated that the binding energy of adsorbates on carbon surfaces is significantly controlled by lattice curvature. Specifically, flat graphene surfaces exhibit a higher physisorption affinity compared to curved carbon nanotubes, suggesting that the convex configuration of fillers may adversely affect the stable residence of gas molecules. Simultaneously, experimental research by Gotovac et al. [30] further confirmed that the nanoscale curvature of carbon nanotube surfaces restricts the effective contact area between the adsorbate and the tube wall, resulting in adsorption interactions significantly weaker than those on flat surfaces. This collectively implies that the convex curvature of fillers may pose a disadvantage for the stable capture of gas molecules. On the other hand, the adsorption process is fundamentally governed by the interplay between physical pore structure and surface chemical properties [31]. However, existing research has predominantly focused on optimizing adsorption performance by enhancing physical parameters such as specific surface area or pore volume [32]. For instance, Zou et al. [33] demonstrated that optimizing mesoporous structures through functionalized multi-walled carbon nanotubes (MWCNTs) could increase the specific surface area of aerogels to 580 m^2^/g; furthermore, Karamikamkar et al. [34] confirmed that utilizing graphene nanoplatelets (GnPs) to inhibit structural stacking during the gelation process enables the construction of ultralight aerogels with extremely high specific surface areas. Furthermore, strategic microporous design is now understood to be critical in balancing material toughness and adsorption accessibility [35]. This emphasis on physical volume expansion has led researchers to relatively overlook the regulatory mechanisms of skeletal surface hydrophilicity/hydrophobicity on solid–gas interfacial interaction energy; advanced interface engineering remains a pivotal frontier for achieving stable and efficient capture of target molecules within hybrid porous systems [36]. In particular, the synergistic effect between physical framework construction and chemical surface modification remains insufficiently elucidated [37]. Given that traditional macroscopic adsorption experiments struggle to directly observe pore evolution at the atomic scale or precisely decouple the independent adsorption contributions of the matrix and fillers in composite materials [38], it is particularly essential to employ molecular dynamics simulation methods to explore the intercomponent interactions and adsorption mechanisms from a microscopic perspective.

While significant progress has been achieved in understanding pore regulation in pure silica aerogels and the adsorption characteristics of carbon-based materials, the synergistic adsorption mechanism within carbon nanotube (CNT)-reinforced hybrid silica aerogel systems remains in the exploratory stage. In particular, how filler incorporation induces geometric reconfiguration and subsequently alters the micro-connectivity of the porous framework is not yet fully understood. A primary academic challenge involves maximizing adsorption efficiency through precise interface engineering while simultaneously leveraging carbon nanomaterials to bolster the structural stability of the porous skeleton. Specifically, there is a lack of systematic molecular dynamics evidence to establish a precise nonlinear correlation between CNT doping mass fraction (5–20 wt%) and nitrogen adsorption capacity, which is essential for identifying the performance gain threshold associated with local filler agglomeration and steric hindrance effects. Furthermore, the competitive mechanism between micropore filling and mesopore condensation at heterogeneous interfaces, as well as the specific impact of potential priority transport channels constructed by CNTs on diffusion kinetics, has not been clearly elucidated from a physical perspective. Based on the microstructural design principles of porous composite materials, the motivation for concurrently incorporating both CNTs and graphene into the silica matrix in this study extends beyond simulating a generic empirical reinforcement strategy. Instead, it is fundamentally designed to investigate synergistic adsorption effects and establish an explicit internal geometric control. By introducing graphene as an idealized two-dimensional flat reference within the same confined porous environment, we can directly compare its adsorption behavior against the continuous convex curvature of the one-dimensional CNTs. This controlled co-doping approach enables a rigorous mechanistic evaluation of how the specific nanoscale curvature of rigid reinforcements dictates solid–gas interfacial interactions and overall gas capture efficiency. Therefore, this study aims to address these knowledge gaps by developing all-atom models to quantitatively analyze the regulatory effects of CNT content on adsorption energy distribution and transport pathways, providing microscopic molecular reconstruction guidance for the design of novel composite materials that simultaneously exhibit exceptional mechanical robustness and superior adsorption performance.

In view of these challenges, this study employs all-atom molecular dynamics (MD) simulations to construct composite pore models comprising silica aerogels synergistically doped with carbon nanomaterials of distinct topological structures, namely carbon nanotubes (CNTs) and graphene. The motivation for concurrently incorporating both carbon nanotubes (CNTs) and graphene into the silica matrix is to investigate synergistic adsorption effects and establish an internal geometric control. By using graphene as an idealized flat reference, we can precisely decouple the impact of the continuous convex curvature of CNTs on the nitrogen adsorption mechanism. The primary objective is to precisely decouple the differential contributions of the curvature effects of rigid fillers and surface chemical properties to gas adsorption behavior at the microscopic scale. By comparatively analyzing the adsorption conformations, density distributions, and diffusion kinetics of nitrogen molecules on flat graphene surfaces versus the curved outer walls of CNTs, we comprehensively elucidate the synergistic adsorption mechanisms at the matrix-filler interface. Furthermore, this work quantitatively evaluates the trade-off between maintaining the mechanical robustness of the skeletal framework and maximizing adsorption capacity across different carbon nanostructures. The findings are intended to provide explicit microscopic theoretical guidance for the design of next-generation composite aerogels that simultaneously exhibit superior mechanical robustness and exceptional adsorption performance.

## 2. Results

### 2.1. Verification of Simulation System Equilibrium

**Figure 1:**
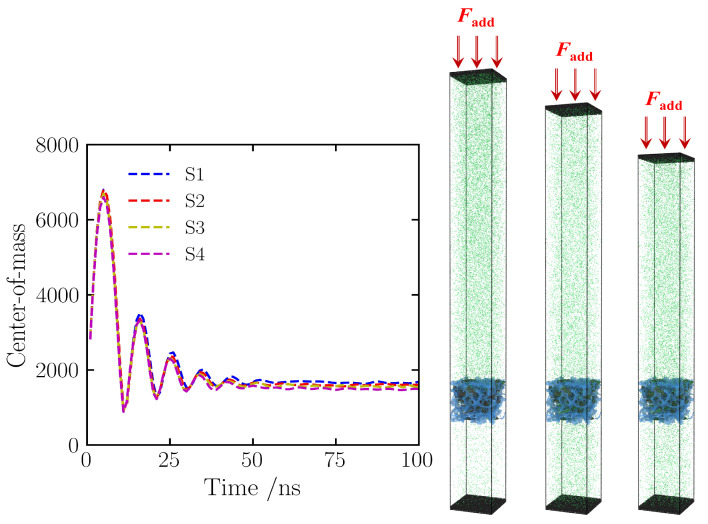
The variation of the center of mass height of the upper plate with simulation time. The red arrows indicate the applied external force ($F_{add}$).

Achieving thermodynamic equilibrium is a prerequisite for performing effective statistical mechanical sampling and accurately assessing adsorption performance. Given that this study employs the piston method to regulate pressure within the NPT ensemble, the vertical displacement characteristics of the center of mass (COM) of the upper rigid plate serve as a definitive criterion to determine whether the system has attained volumetric equilibrium and adsorption saturation [39]. Figure 1 illustrates the evolutionary trajectory of the upper plate’s COM height over the course of the simulation. During the initial phase (0–20 ns), the COM height exhibits a rapid descending trend, corresponding to the process where nitrogen molecules, driven by pressure, swiftly populate the porous skeleton, thereby inducing volumetric shrinkage of the system. Subsequently (t > 40 ns), the position curve of the COM gradually converges and fluctuates with minor amplitudes around a specific height due to thermal fluctuations, exhibiting no further monotonic drift over time. This indicates that under the set pressure of 5 atm, following 100 ns of relaxation, gas molecules have completed their diffusion and filling within the pores, and the system has macroscopically reached a dynamic equilibrium state of adsorption–desorption. Consequently, to ensure the reliability of the data, all subsequent statistics regarding adsorption capacity and kinetic analyses were derived by sampling equilibrium data from the final 50 ns of the simulation trajectory. Given the massive scale of the all-atom systems (>230,000 atoms), statistical reliability was ensured via a long 50 ns equilibrium sampling period. According to the Ergodic hypothesis, the time-average of such a large and fully equilibrated system effectively represents an ensemble average. To verify reproducibility, intrinsic statistical fluctuations were quantified using block averaging and are presented as error bars in all data plots.

### 2.2. Impact of Carbon Nanotube Doping

#### 2.2.1. Overall Adsorption Performance of Silicon-Based Porous Materials

**Figure 2:**
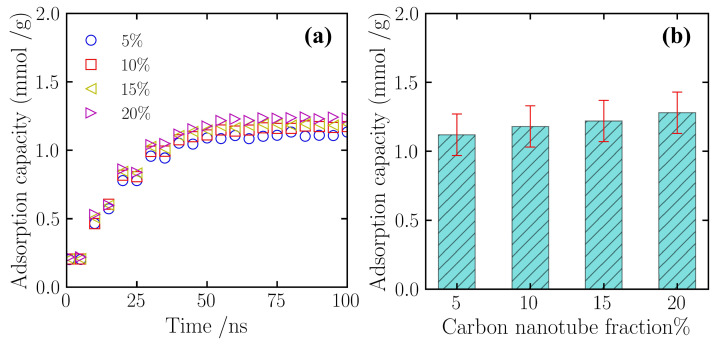
The overall nitrogen adsorption capacity of the composite porous materials under varying CNT mass fractions (5%, 10%, 15%, and 20%). (**a**) Dynamic evolution of adsorption capacity over the 100 ns simulation time. (**b**) Comparison of the final equilibrium adsorption capacities. All simulations were conducted in the NPT ensemble at T = 300 K and P = 5 atm. Error bars represent the standard deviation derived from the equilibrium sampling over the final 50 ns.

This section focuses on the macroscopic influence of carbon nanotube (CNT) doping levels on the overall nitrogen adsorption capacity of silicon-based porous materials. A series of composite material models featuring varying CNT mass fractions (5%, 10%, 15% and 20%) were constructed for this investigation. Figure 2a illustrates the dynamic evolution of nitrogen adsorption capacity over the simulation time across different operating conditions. Observation of the adsorption kinetics curves reveals that during the initial simulation phase (0–25 ns), all scenarios exhibited extremely rapid adsorption rates. In this stage, the systems achieved approximately 70–80% of their total adsorption saturation, reflecting the rapid ingress of nitrogen molecules into the effective internal pores driven by concentration gradients. Subsequently, as adsorption sites were progressively occupied, the adsorption rate plateaued. After approximately 50 ns, the curves for all systems entered a stable plateau region, with adsorption capacities fluctuating slightly within the range of 1.12–1.28 mmol/g, indicating that the systems had attained a dynamic adsorption–desorption equilibrium.

Figure 2b further compares the final average adsorption capacities of each simulation system upon reaching equilibrium. Quantitative analysis indicates that within the studied range of doping mass fractions (5–20%), the equilibrium adsorption capacity of the silicon-based porous material for nitrogen exhibits a monotonically increasing trend. As depicted, at a CNT doping level of 5%, the equilibrium adsorption capacity of the system was approximately 1.12 mmol/g. With increasing doping content, the adsorption capability improved steadily: capacities for the 10% and 15% doping scenarios rose to approximately 1.18 mmol/g and 1.22 mmol/g, respectively. When the CNT mass fraction reached 20%, the equilibrium adsorption capacity of the composite peaked at approximately 1.28 mmol/g. Notably, compared to the low-doping condition of 5%, the nitrogen adsorption capacity of the 20% high-doping system demonstrated an overall enhancement of approximately 15%.

#### 2.2.2. Adsorption of the Silica Aerogel Component

**Figure 3:**
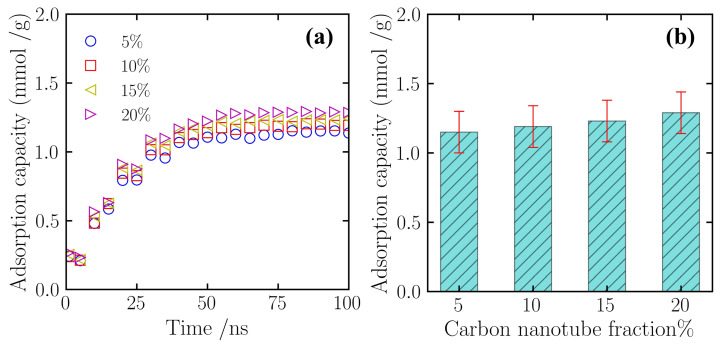
Adsorption capacity of the silica aerogel component at different CNT doping levels. (**a**) The variation of adsorption capacity over time; (**b**) Comparison of equilibrium adsorption capacity. Data were collected under constant temperature (300 K) and pressure (5 atm) conditions.

To delve deeper into the intrinsic source of the adsorption enhancement effect arising from CNT incorporation, this section independently analyzes the adsorption behavior of the silica aerogel framework component within the composite material. Figure 3a illustrates the evolution of nitrogen adsorption capacity for the silica component over simulation time at different CNT doping mass fractions (5%, 10%, 15%, and 20%). As depicted, the silica framework exhibits adsorption characteristics similar to those of the overall material: during the initial 0–30 ns of the simulation, the adsorption capacity shows a rapid growth trend; the rate of increase then slows as adsorption sites become progressively saturated. After the simulation time exceeds 50 ns, the adsorption capacity curves for all cases enter a stable plateau, indicating that the silica aerogel framework has reached a dynamic adsorption–desorption equilibrium.

By statistically analyzing the number of adsorbed molecules in the equilibrated simulation systems, the equilibrium nitrogen adsorption capacity of the silica framework under different CNT doping levels was obtained. Figure 3b shows that as the total CNT doping mass fraction in the silicon-based porous material increases, the nitrogen adsorption capacity of the silica matrix component exhibits a progressively increasing trend. At the 5% doping scenario, the equilibrium adsorption capacity of the silica component was 1.15 mmol/g. As the doping concentration increased, this value rose to 1.19 mmol/g and 1.23 mmol/g for the 10% and 15% scenarios, respectively. When the doping level reached 20%, the adsorption capacity of the silica framework was 1.29 mmol/g. These results indicate that the incorporation of carbon nanotubes, to some extent, improves the nitrogen adsorption performance of the silica aerogel matrix.

#### 2.2.3. Adsorption of the Graphene Sheets Component

**Figure 4:**
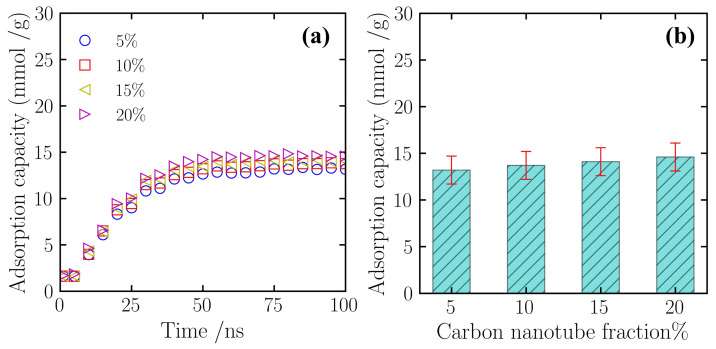
Adsorption capacity of the graphene sheets component at different CNT doping levels. (**a**) The variation of adsorption capacity over time; (**b**) Comparison of adsorption capacity under different operating conditions. Data were collected under constant temperature (300 K) and pressure (5 atm) conditions.

To comprehensively evaluate the adsorption contribution of each component in the composite material, this section further analyzes the nitrogen adsorption behavior of the graphene sheets at different CNT doping concentrations. Figure 4a presents the variation of the adsorption capacity of the graphene component over simulation time. Observation of the kinetic curves for each scenario reveals that the adsorption trajectories for graphene exhibit similar trends across the different doping systems. In the first 30 ns of the simulation, the adsorption capacity increases relatively quickly, after which the rate gradually slows. When the simulation time reaches 50 ns, the adsorption capacity in all systems enters a dynamic equilibrium phase. The numerical values of the adsorption curves for each scenario are quite close, indicating that the introduction of CNTs did not significantly interfere with the adsorption kinetic characteristics of the graphene surface.

Figure 4b further quantifies the average equilibrium adsorption capacity of the graphene component for each scenario. In the 5% CNT doping scenario, the adsorption capacity of the graphene sheets was 13.2 mmol/g. As the CNT doping mass fraction increased, this value exhibited a slight upward trend. When the doping levels were 10% and 15%, the adsorption capacities changed to 13.8 mmol/g and 14.2 mmol/g, respectively. By the 20% doping scenario, the adsorption capacity was 14.7 mmol/g. Although the adsorption capacity shows a slight increase with doping concentration, the overall magnitude of change is relatively small; considering the margin of error, this suggests that in this simulation system, the introduction of carbon nanotubes has a relatively limited impact on the intrinsic adsorption performance of the graphene component itself.

#### 2.2.4. Adsorption of the Carbon Nanotubes Component

**Figure 5:**
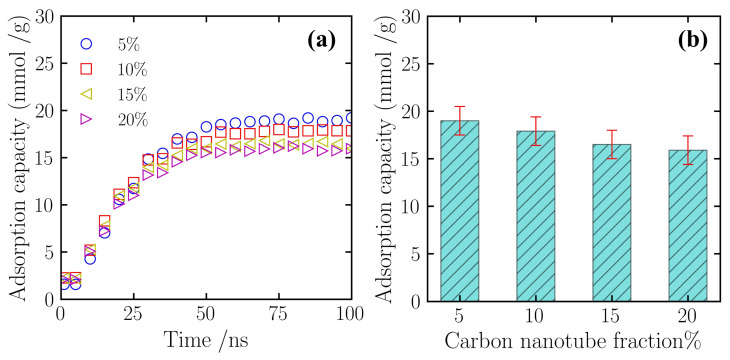
Adsorption capacity of the carbon nanotubes component at different doping levels. (**a**) The variation of adsorption capacity over time; (**b**) Comparison of adsorption capacity under different operating conditions. Data were collected under constant temperature (300 K) and pressure (5 atm) conditions.

To clarify the direct adsorption contribution of the carbon nanotube fillers in the composite material, this study conducted an independent analysis of their own nitrogen adsorption behavior. Figure 5a displays the trajectories of nitrogen adsorption capacity for this component over simulation time at different CNT doping concentrations. Observation of the curves shows that the carbon nanotubes in all scenarios exhibit consistent adsorption kinetic characteristics. In the initial stage of the simulation, from 0 to 30 ns, nitrogen molecules rapidly adsorb onto the CNT surfaces, with the adsorption capacity showing a relatively steep increasing trend over time. Subsequently, the adsorption rate gradually slows as the surface sites become occupied. After 50 ns of simulation time, the adsorption capacity values for each system remain at a relatively constant level with minor fluctuations, signifying that the carbon nanotube component has reached a state of thermodynamic adsorption equilibrium.

Statistical analysis of the equilibrium data reveals the relationship between the adsorption capacity of the CNT component and the doping concentration. Figure 5b shows that as the total doping mass fraction of CNTs in the system increases from 5% to 20%, the corresponding specific equilibrium adsorption capacity (per unit mass) shows a decreasing trend. As depicted, in the 5% doping scenario, the equilibrium adsorption capacity of the CNTs was 19.0 mmol/g. As the doping proportion increased, this value gradually decreased, reaching 17.9 mmol/g and 16.5 mmol/g in the 10% and 15% scenarios, respectively. When the doping level reached 20%, the adsorption capacity dropped to 15.8 mmol/g. This result indicates that in the current simulation system, while increasing the doping level enhances the overall adsorption performance of the composite material, the specific adsorption efficiency (per unit mass) of the carbon nanotubes themselves shows a declining trend.

### 2.3. Impact of Surface Hydrophilicity/Hydrophobicity on Adsorption

After clarifying the influence of CNT doping concentration, a physical structural factor, this study further investigates the regulatory role of surface chemical properties on adsorption behavior. To simulate material surfaces with different hydrophilic/hydrophobic characteristics, a scaling factor, λ, was introduced to adjust the Lennard-Jones potential energy parameters between the atoms of the solid framework and the gas atoms. This method, based on classical molecular dynamics theory, linearly modulates the van der Waals interaction strength at the solid–gas interface and has been widely used to characterize the hydrophilic or hydrophobic wetting properties of material surfaces [40]. To ensure a rigorous comparative analysis and eliminate interference from other variables, the model with a 5% CNT mass fraction was selected as the baseline condition. On this basis, a total of five sets of simulation experiments were established, with λ values of 0.3, 0.8, 1.0, 1.2, and 1.5, respectively. Here, a λ value less than 1.0 represents a weakly interacting hydrophobic state, while a value greater than 1.0 represents a hydrophilic state with enhanced interaction.

**Figure 6:**
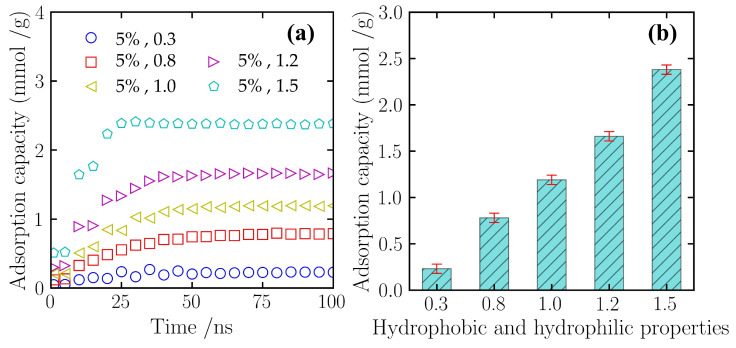
Effect of surface hydrophilicity/hydrophobicity (controlled by the potential scaling factor λ) on the nitrogen adsorption capacity of the silicon-based porous materials. (**a**) Time-dependent adsorption kinetics curves. (**b**) Comparison of equilibrium adsorption capacities across different solid–gas interfacial energy states. Simulations were performed at 300 K and 5 atm, with the 5% CNT doping scenario serving as the structural baseline.

This study employed the established adsorption identification algorithm, using a cutoff radius of 1.5 nm, to identify and quantify the adsorbed nitrogen molecules within the simulation system. Figure 6a illustrates the dynamic evolution of nitrogen adsorption capacity over simulation time under different surface hydrophilicity/hydrophobicity settings. As shown, the change in the surface potential parameter λ influenced the adsorption kinetics. Under the hydrophobic condition of λ = 0.3, the adsorption curve remained at a low level throughout the simulation period, indicating minimal nitrogen molecule adsorption on this surface. Conversely, as the λ value increased to 1.5 (hydrophilic state), the adsorption capacity grew relatively rapidly during the initial 0–20 ns of the simulation and subsequently reached a higher adsorption plateau. Although the numerical values of adsorption capacity differed across the conditions, all systems entered a stable adsorption–desorption dynamic equilibrium state after 50 ns.

Figure 6b further compares the average adsorption capacities of each system after reaching equilibrium. The statistical results show that as the scaling factor λ increases—that is, as the surface hydrophilicity is enhanced—the equilibrium adsorption capacity of the material exhibits an upward trend. Under the strongly hydrophobic condition (λ = 0.3), the equilibrium adsorption capacity was only 0.23 mmol/g, a decrease of approximately 80.7% compared to the baseline condition (λ = 1.0). As λ returned to 1.0, the adsorption capacity recovered to 1.19 mmol/g. When the hydrophilicity was further enhanced to λ = 1.5, the equilibrium adsorption capacity increased to 2.38 mmol/g. Compared to the baseline condition, the adsorption capacity of the strongly hydrophilic surface was enhanced by approximately 98%. This result indicates that the surface hydrophilic/hydrophobic properties of silicon-based porous materials are a critical factor influencing the adsorption behavior of gas molecules on the pore surfaces.

## 3. Discussion

To provide a profound insight into the adsorption and diffusion behaviors of nitrogen within silicon-based porous composites synergistically doped with carbon nanotubes (CNTs) and graphene at the micro- and nanoscale, molecular dynamics models were developed and subjected to prolonged simulations. After a relaxation period of 100 ns, all simulation systems attained a stable adsorption–desorption equilibrium. Adsorption capacity data were acquired by statistically quantifying the nitrogen molecules captured by the porous framework in the equilibrated models, employing the adsorption identification algorithm detailed in Section 2.2. This section presents the simulation results, beginning with a quantitative analysis of the specific impact of different CNT doping mass fractions on the adsorption performance of the composite material and its individual constituents. Subsequently, the influence of surface hydrophilicity and hydrophobicity on nitrogen adsorption behavior is further characterized. Finally, by integrating macroscopic and microscopic simulation evidence, the underlying adsorption enhancement mechanisms intrinsic to the silicon-based porous materials are discussed.

### Discussion of Adsorption Mechanism

**Figure 7:**
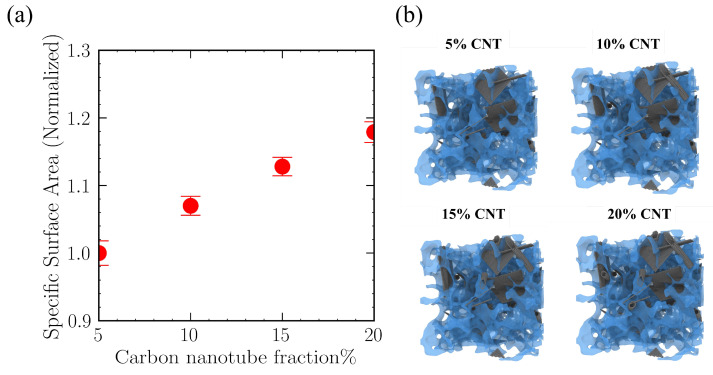
Impact of carbon nanotube doping levels on the geometric characteristics of the porous material. (**a**) Variation of normalized specific surface area; (**b**) Three-dimensional visualization of the material structure.

The simulation results indicate that the enhancement of the overall adsorption performance of the silicon-based porous material and the silica matrix by CNT doping is primarily attributed to the geometric reconfiguration of the porous structure by the nanofillers. The three-dimensional visualization in Figure 7b shows that the rigid CNT network constructs a supporting skeleton within the matrix, which, to some extent, alleviates the densified packing of the amorphous silica aerogel during the relaxation process. To quantify this change in geometric characteristics, this study calculated the normalized specific surface area for different doping concentrations. As shown in Figure 7a, the total effective specific surface area of the material exhibits an increasing trend with the increase in CNT doping mass fraction. The calculation results show that, compared to the 5% doping system, the specific surface area in the 20% doping scenario increased by an overall 18.25%. The increase in specific surface area enhances the contact probability between nitrogen molecules and the solid framework, thereby providing more physical adsorption sites.

Beyond mere geometric expansion, the physical presence of these rigid nanofillers profoundly alters the microenvironment for gas capture by modulating both the diffusion pathways and the thermodynamic quality of the adsorption sites. Geometrically, the dispersed CNTs and graphene reshape the mesoporous network into highly tortuous confined channels. This nanoconfinement effect restricts the translational degrees of freedom and the mean free path of the nitrogen molecules, fundamentally altering their macroscopic diffusion pathways. Consequently, the increased structural tortuosity increases their collision frequency with the skeleton and prolonging their local residence time. Thermodynamically, the sp^2^-hybridized carbon networks generate intense overlapping van der Waals potential fields, particularly at the carbon-silica boundaries. As demonstrated by previous high-resolution molecular dynamics studies on carbon-doped porous media [41], fluid molecules exhibit pronounced localized densification immediately adjacent to rigid carbon surfaces. Therefore, the observed non-linear enhancement in macroscopic adsorption capacity is fundamentally driven by this synergistic mechanism: the structural tortuosity of the mesoporous network combined with the creation of high-affinity interfacial energy traps. These molecular-level predictions exhibit strong qualitative alignment with established experimental trends. For instance, Sun et al. [35] demonstrated that CNT incorporation significantly expanded the pore volume of silica aerogels from 2.14 cm^3^/g to 2.92 cm^3^/g, leading to a marked increase in nitrogen uptake. This empirical evidence of physical structural expansion and enhanced adsorption capacity validates the 18.25% surface area enhancement and 15% overall capacity increase predicted by our MD models.

It is noteworthy that although the overall adsorption performance of the composite material improved with increasing doping levels, the specific adsorption efficiency (per unit mass) of the CNT component itself exhibits a declining trend (Section 2.2.4). This phenomenon is attributed to the unique microscopic geometric configuration of CNTs and their aggregation behavior at high concentrations. From the perspective of individual geometry, our group’s previous research confirmed^8^ that the geometric flatness of carbon material surfaces significantly affects gas adsorption capacity; when the wrinkle angle on a graphene surface exceeds 20°, its adsorption capacity is significantly lower than that of flat graphene. Geometrically, a carbon nanotube can be regarded as a rolled-up graphene sheet with a constant convex curvature. Compared to a flat graphene structure, this continuous convex configuration is unfavorable for the stable residence of nitrogen molecules [42], which is the intrinsic reason for its lower specific adsorption capacity compared to graphene. Furthermore, as the CNT doping concentration in the system progressively increased from 5% to 20%, constrained by the nanoscale pore space, the high density of filler distribution inevitably led to local stacking and agglomeration. When carbon nanotubes come into contact or intersect with each other, the steric hindrance between the tube walls creates a shielding effect, causing parts of the outer surface that were originally available for adsorption to become inaccessible to gas molecules [43]. This indicates that, in the silicon-based porous composite system, CNTs primarily play a role in structural support and interface modulation, rather than directly serving as high-efficiency adsorption sites.

Building on the optimization of the physical structure, this study further confirms that surface hydrophilic/hydrophobic properties have a significant regulatory influence on adsorption performance. The quantitative analysis in Section 2.3 shows that by adjusting the scaling factor λ to alter the van der Waals interaction strength at the solid–gas interface, it is possible to directly influence the residence capability of nitrogen molecules on the pore surfaces. The data show that when the surface is in a strongly hydrophobic state (λ = 0.3), the equilibrium adsorption capacity is only 0.23 mmol/g, whereas when it is converted to a strongly hydrophilic state (λ = 1.5), the adsorption capacity increases to 2.38 mmol/g. Furthermore, compared to the baseline condition (λ = 1.0), the enhancement in adsorption capacity brought about by the strong hydrophilic modification is approximately 98%. This value is higher than the gain in specific surface area (approximately 18.25%) achieved solely through the physical support of CNTs. This comparison indicates that while the expansion of the physical structure determines the upper limit for the number of adsorption sites, the surface chemistry dictates the effective utilization rate of these sites. Therefore, the ideal design strategy for high-performance adsorption materials should involve an organic synergy between constructing a physical framework with a high specific surface area and tuning the surface to be strongly hydrophilic, in order to maximize adsorption capacity.

These findings provide practical design guidelines for aerogels in applications such as gas storage and environmental remediation. For gas storage scenarios requiring structural durability under pressure cycles, the role of CNTs should be prioritized as a mechanical scaffold to preserve pore volume, rather than as a direct adsorption medium, given their declining specific efficiency at high concentrations. Conversely, for applications prioritizing total capacity, such as gas separation or pollutant capture, our results suggest that surface chemistry engineering is a more effective lever than purely increasing filler content. Specifically, leveraging the high-porosity framework provided by carbon fillers and subsequently optimizing the interfacial affinity through surface functionalization represents a superior strategy for maximizing adsorption performance in complex engineering environments.

## 4. Conclusions

In this study, molecular dynamics simulations were employed to construct a complex model of a silicon-based porous material synergistically doped with carbon nanotubes (CNTs) and graphene. Through an extended 100 ns relaxation simulation, the adsorption behavior and microscopic mechanism for nitrogen were systematically investigated. A quantitative comparative analysis of nitrogen adsorption under different doping conditions and surface characteristics yielded the following main conclusions:

(1) The incorporation of carbon nanotubes had a positive impact on the overall nitrogen adsorption capacity of the silicon-based porous composite material. Within the studied concentration range, as the CNT doping mass fraction increased from 5% to 20%, the overall equilibrium adsorption capacity of the composite material exhibited a monotonically increasing trend, with the total adsorption capacity enhancing by approximately 15%. Microscopic mechanism analysis revealed that the CNT network acted as a skeletal support within the composite system, partially alleviating the densified packing of the amorphous silica aerogel. Structural characterization further confirmed that, compared to the low-doping system of 5%, the normalized specific surface area of the material in the 20% doping scenario increased by 18.25%. This increase in specific surface area enhanced the contact probability between gas molecules and the solid framework, thereby increasing the number of effective sites for physical adsorption.
(2) A comparison of the adsorption performance of individual components revealed a non-linear enhancement mechanism. The increase in overall adsorption capacity was primarily attributed to the enhanced adsorption of the silica aerogel component. Conversely, the specific adsorption efficiency (per unit mass) of the CNT component itself showed a declining trend with increasing doping concentration. In conjunction with theoretical analysis, this phenomenon is attributed to two factors: on one hand, the inherent convex curvature of the CNTs results in a relatively shallow solid–gas interaction potential well, which is unfavorable for the stable residence of nitrogen molecules; on the other hand, local stacking and agglomeration of the fillers at high doping concentrations produced a steric hindrance and shielding effect, leading to a loss of some effective adsorption surface area. This result indicates that CNTs in the composite system primarily serve to maintain the skeletal structure and modulate the pore distribution, rather than acting directly as high-efficiency adsorption sites.
(3) Surface hydrophilic/hydrophobic properties play a significant regulatory role in adsorption performance. The simulation results showed that when the surface was adjusted to a hydrophilic state (λ = 1.5) by modulating the potential energy parameters, the equilibrium nitrogen adsorption capacity increased by approximately 98% relative to the baseline condition (λ = 1.0). This enhancement is significantly higher than the gain in specific surface area (approximately 18.25%) achieved through CNT doping. This data comparison indicates that while the physical framework provides the foundation for the number of adsorption sites, the surface chemistry dictates the efficiency of site utilization. Therefore, combining a physical framework with a high specific surface area with hydrophilic surface modification is a viable strategy for enhancing the adsorption performance of such composite materials.

In summary, the broader scientific implication of this study lies in elucidating the distinct roles of physical structural scaffolding and interfacial thermodynamics in hybrid aerogel systems. The findings demonstrate that while rigid carbon nanofillers are essential for preserving the free volume of the mesoporous network against densification, their intrinsic surface geometry limits direct adsorption efficiency. This mechanistic insight provides a rational, decoupled design strategy for future advanced porous materials. To achieve optimal performance, future engineering efforts should utilize carbon nanomaterials primarily to construct stable mechanical frameworks, while simultaneously relying on specific surface chemical functionalizations (e.g., maximizing polarity or hydrophilicity) to generate high-affinity adsorption sites. This objective theoretical guidance is expected to facilitate the development of more efficient and mechanically stable composite aerogels for practical gas capture and storage applications.

## 5. Materials and Methods

In this study, all-atom molecular dynamics (MD) simulations were employed to systematically investigate the adsorption and diffusion behaviors of nitrogen (N_2_) within silicon-based porous composites doped with carbon nanotubes (CNTs) and graphene at the micro- and nanoscale. All simulation procedures were executed using the open-source software package LAMMPS (version 23 June 2022) [44].

### 5.1. Molecular Dynamics Model Construction

**Figure 8:**
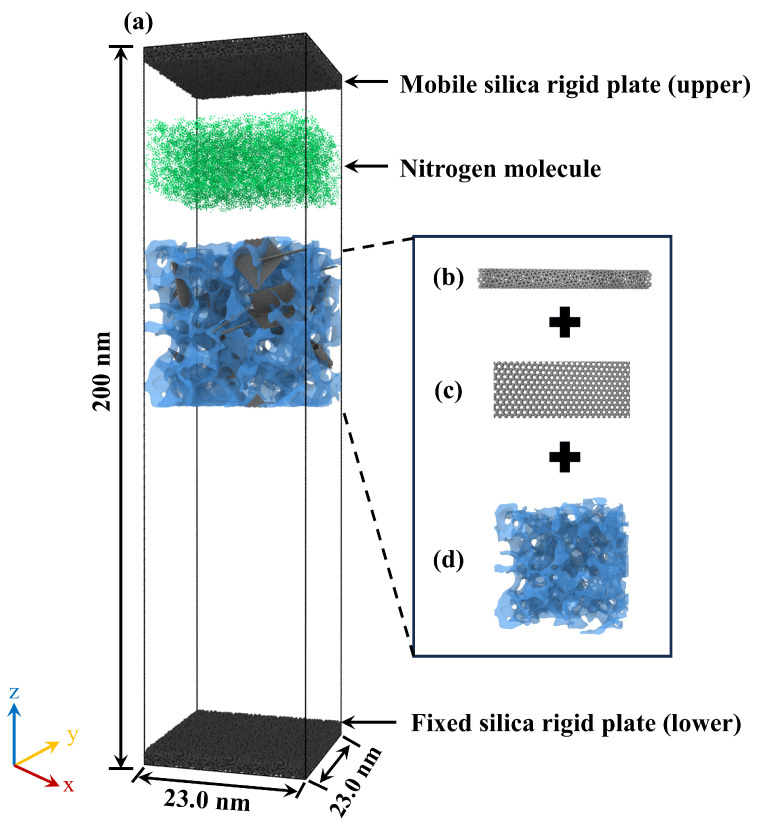
Molecular dynamics simulation system. (**a**) Molecular model; (**b**) Carbon nanotubes; (**c**) Graphene sheets; (**d**) Silica aerogel framework.

To elucidate the impact of synergistic doping with carbon nanotubes (CNTs) and graphene on the nitrogen adsorption behavior of silicon-based porous materials at the micro- and nanoscale, a series of all-atom molecular dynamics (MD) models were constructed using the Large-scale Atomic/Molecular Massively Parallel Simulator (LAMMPS). Four distinct simulation scenarios were designed with varying CNT doping mass fractions of 5%, 10%, 15%, and 20%, respectively. The selected CNT mass fractions (5–20%) represent a realistic experimental window for reinforced silica aerogels. According to established experimental literature [45,46], filler loadings within the 1–15 wt% range are typically utilized to achieve optimal mechanical enhancement while preserving high porosity. The upper limit of 20% was intentionally chosen in this study to investigate the structural stability and adsorption performance limits near the physical saturation threshold, beyond which significant CNT agglomeration and pore blockage often occur in practical synthesis. This concentration gradient allows for a systematic evaluation of how filler density dictates the non-linear evolution of adsorption energy distributions and gas diffusion kinetics. As illustrated in Figure 8a, the dimensions of the simulation domain were established as 28.6 × 14.3 × 200.0 nm^3^, it is important to note that the highly elongated Z-axis dimension (200.0 nm) is not entirely occupied by the solid matrix. Instead, it is specifically designed to incorporate a massive vacuum space above the aerogel framework, serving as a “gas reservoir”. This allows the mobile rigid plate sufficient travel distance to act as a piston, continuously compressing the nitrogen molecules to maintain a constant 5 atm pressure without depleting the free gas phase. The molecular dynamics model comprises the following critical components: (1) two rigid atomic plates positioned along the Z-axis for system pressure regulation, where the lower plate remains fixed while the upper plate is mobile along the *Z*-axis to modulate internal pressure; (2) the silicon-based porous material; and (3) nitrogen molecules. Specifically, the silicon-based porous material is constituted by an amorphous silica aerogel acting as the skeletal matrix, co-doped with graphene sheets (comprising 32,000 carbon atoms) and carbon nanotubes. The silica matrix was generated via a melt-quench protocol (7000 K to 300 K) combined with the negative pressure rupturing method to ensure a realistic porous network. Specifically, the matrix comprises 192,000 atoms (64,000 Si and 128,000 O) within a cubic volume of 230.71 Å^3^, achieving a target bulk density of 0.520 g/cm^3^. The structural connectivity of the amorphous skeleton was verified via Radial Distribution Function (RDF) analysis, yielding characteristic Si-O, Si-Si, and O-O bond lengths of 1.61, 3.07, and 2.63 Å, respectively^8^ [8]. This structural configuration naturally provides a highly interconnected mesoporous network with pore sizes primarily distributed around 2.0 nm. CNTs (length ~10.1 nm) and graphene (~60 × 67 Å) were randomly incorporated into the silica matrix with no preferred orientation or covalent bonding. Interfacial interactions were governed strictly by non-bonded physical forces, enabling the decoupling of filler topological effects on adsorption.

The silica aerogel framework (Figure 8d) was generated via the classical melt-quench simulation protocol [47]. Specifically, a crystalline precursor containing a predetermined number of silicon and oxygen atoms was melted at elevated temperatures, subsequently quenched to room temperature at a rapid cooling rate, and allowed to fully relax, thereby yielding a vitreous model characterized by a disordered network structure. Upon this matrix, pre-optimized graphene (Figure 8b) and carbon nanotube (Figure 8c) fillers were further incorporated to finalize the construction of the silicon-based porous composite material.

In all simulation systems, the atomic count for each constituent was precisely calibrated to satisfy specific experimental conditions. Each model consistently contained 110,446 silica molecules. The number of carbon atoms constituting the graphene sheets was maintained at a constant value of 30,000 across all scenarios. The quantity of nitrogen molecules, serving as the adsorbate, was set to 20,000. Regarding the carbon nanotube fillers, four distinct simulation cases were established to investigate the impact of varying doping concentrations on material performance. Table 1 details the geometric models of the silicon-based porous materials and their corresponding carbon atom counts under different CNT doping mass fractions (5%, 10%, 15%, and 20%). As the doping level increased, the total number of carbon atoms within the CNTs was 6300, 12,600, 18,900, and 25,200, respectively. To simulate bulk material properties and eliminate finite-size surface effects, periodic boundary conditions were applied along all three spatial dimensions for every simulation box [48]. Furthermore, the rationale for concurrently deploying both CNTs and graphene within the simulation models is to establish an explicit internal geometric control. In this setup, the graphene sheets serve as a baseline flat surface, allowing for a rigorous comparative analysis against the highly curved exterior walls of the CNTs. This experimental design systematically decouples the impact of filler topology and surface curvature on the dynamic adsorption behavior of nitrogen molecules, ensuring that the observed differences in adsorption capacity can be directly attributed to the geometric configurations of the respective carbon nanostructures.

### 5.2. Force Field Parameters and Interaction Potentials

The accurate description of interatomic interaction potential functions is pivotal for ensuring the reliability of molecular dynamics simulation outcomes. Within the simulation system, the internal C-C bonding interactions for carbon atoms residing in graphene sheets and carbon nanotubes are characterized using the AIREBO potential [49]. This potential function is capable of effectively simulating the breaking and formation of covalent bonds, as well as the diverse hybridization states inherent to carbon materials. The interactions involving nitrogen molecules are modeled using short-range Van der Waals forces (*U*_VdW_) and long-range Coulombic interactions (*U*_Coul_), expressed as follows:

(1) $$U_{\mathrm{UdW}}=\sum_{i}\sum_{j>i}\epsilon_{ij}\left[{\left(\frac{\sigma_{ij}}{r_{ij}}\right)}^{12}-{\left(\frac{\sigma_{ij}}{r_{ij}}\right)}^{6}\right]$$

(2) $$U_{\mathrm{Coul}}=\frac{q_{i}q_{j}}{4\pi \epsilon_{0}r_{ij}}$$

Here, *U*_VdW_ and *U*_Coul_ denote the short-range Van der Waals forces and Coulombic interactions, respectively, while *r*_ij_ and *ϵ*_ij_ represent the relative distance and energy parameter between atoms i and j. To ensure physical consistency and dimensional accuracy, all molecular dynamics simulations were strictly executed using the metal unit system in LAMMPS, where energy, distance, and time are expressed in electron-volts (eV), Angstroms (Å), and picoseconds (ps), respectively. For the long-range Coulombic calculations, a dimensionless relative dielectric constant of $\epsilon_{r}=1.0$ was employed. The variables $q_{i}$ and $q_{j}$ correspond to the partial atomic charges. To strictly maintain the macroscopic electroneutrality of the system, the silicon atoms were assigned a positive charge of $q_{Si}=+0.6\;e$, and the oxygen atoms were assigned a negative charge of $q_{O}=-0.3e$, yielding a neutral SiO_2_ framework. Carbon atoms within the graphene and carbon nanotube structures are treated as electrically neutral, with a charge of 0 e. For the nitrogen molecular model, the nitrogen atoms carry a charge of −0.482 e, and the central massless site bears a charge of 0.964 e. The interactions within the silica matrix are described using the Tersoff potential, which has been widely proven effective in accurately reproducing the Si-O bond-order and short-range structural features during the melt-quench generation of amorphous networks. Furthermore, because the capture of nitrogen molecules within the hybrid aerogel is fundamentally a physical adsorption (physisorption) process driven by dispersion forces, all solid–gas interfacial interactions (e.g., silica-nitrogen and carbon-nitrogen) are strictly modeled via short-range Van der Waals forces using the standard Lennard-Jones (LJ) potential [50]. This LJ-based approach is a widely accepted and successful standard for characterizing physical adsorption kinetics in complex porous media [51]. Detailed parameter configurations are provided in Table 2 [8]. To simulate variations in surface chemistry (i.e., hydrophilic versus hydrophobic conditions), the well depth parameter (ϵ) of the solid-fluid Lennard-Jones potential was systematically scaled based on a “fluid-like” adjustment strategy. Physically, this parameter acts as a thermodynamic proxy for the material’s surface energy state, directly modulating the work of adhesion ($W_{a}$) at the solid–gas interface. Decreasing ϵ mimics the experimental grafting of low-surface-energy non-polar groups (e.g., methyl groups via HMDS modification), which shields the high-energy skeleton and results in a hydrophobic state. Conversely, increasing ϵ represents the presence of high-density polar silanol (-OH) or carboxyl groups, yielding a strongly hydrophilic surface [28]. This abstraction effectively maps complex surface functionalizations to a unified thermodynamic quantity, enabling the quantitative decoupling of surface energy effects from the geometric features of the porous network.

### 5.3. Simulation Details

To systematically investigate the adsorption and diffusion behaviors of nitrogen molecules within the silicon-based porous materials at the micro- and nanoscale, extensive computations were conducted using the molecular dynamics simulation software LAMMPS (2 August 2023 version). The molecular simulation process was executed within the isothermal-isobaric (NPT) ensemble, employing a relaxation period of 100 ns to ensure the system was fully equilibrated and reached a state of adsorption equilibrium. The temperature of the simulation system was maintained constant at 300 K using the Nosé-Hoover thermostat, while the pressure was regulated at 5 atm via the piston pressure control method [8].

The piston pressure control method conceptualizes the movable rigid plate (upper boundary) as a piston, regulating the system’s pressure through its vertical displacement. In accordance with the designated simulation pressure (5 atm), a constant external force (Fadd) is applied to the rigid control plate:

(3) $${P=F}_{\mathrm{add}}N_{s}/S$$

where P represents the simulation system pressure (5 atm), Ns denotes the total number of atoms in the rigid plate (53,880), and S is the surface area of the rigid plate.

Consequently, based on Equation (3), the force applied to each atom of the rigid plate is derived as Fadd = 2.4 × 10^−6^ eV/Å. Through this mechanism, the upper plate dynamically adjusts its position along the Z-axis in response to the equilibrium between the internal fluid pressure and the externally applied force, thereby achieving precise control over the system pressure. The equations of motion were integrated using the velocity-Verlet algorithm with a time step of 10 fs. For the Nosé-Hoover thermostat, a temperature damping constant ($T_{damp}$) of 1.0 ps was employed to ensure stable thermal equilibrium. Prior to the production phase, the initial structures were optimized via the conjugate gradient (CG) algorithm with a force tolerance of $1.0\times 10^{-7}$ eV/Å to ensure structural stability.

The self-diffusion coefficient (*D*) of nitrogen molecules within the composite porous materials was rigorously derived from the Mean Square Displacement (MSD) using the standard Einstein relation, governed by the following equation [25]:

(4) $$D=\frac{1}{6}\underset{t\to \infty }{\lim}\frac{d}{dt}\langle |r(t)-r(0){|}^{2}\rangle$$

where $\left\langle |r(t)-r(0){|}^{2}\right\rangle$ represents the ensemble-averaged MSD of the gas molecules. To avoid calculation artifacts originating from the initial ballistic motion and terminal statistical fluctuations, the linear regression fitting of the MSD curves was strictly executed within the normal Fickian diffusion regime. Specifically, the linear fitting time interval was designated from 10 ns to 40 ns during the steady-state production phase, ensuring a high linearity ($R^{2}>0.98$). Furthermore, the statistical uncertainty associated with the diffusion results was quantified using the block averaging technique. The total sampling trajectory was segmented into independent blocks to yield multiple slope calculations, with the standard deviation of these independent measurements reported as the final statistical error margins.

### 5.4. Adsorption Identification Algorithm

Adsorption is defined as the phenomenon wherein gas molecules or atoms form a relatively stable aggregated state on a solid surface under the influence of potential fields [52]. In the simulated system, following 100 ns of molecular dynamics simulation, a fraction of the nitrogen molecules are captured by the silicon-based porous material, transitioning into an adsorbed state. Meanwhile, the remaining nitrogen molecules continue to diffuse freely within the pore centers, referred to as the free state. When the system reaches a steady state, the nitrogen adsorption and desorption processes attain dynamic equilibrium (i.e., the number of molecules adsorbed per unit time is substantially equal to the number of molecules desorbed).

To accurately identify and quantify the adsorbed nitrogen molecules, this study employs a recognition algorithm based on a geometric distance cutoff [42]. By iteratively executing this algorithm, the instantaneous count of adsorbed nitrogen molecules within the simulation system is obtained. The algorithm determines whether a specific nitrogen molecule is adsorbed by calculating the relative distance, d, between the molecule’s center of mass (COM) and its nearest neighbor atom in the solid framework. The formula for calculating distance d is given by:

(5) $$d=\sqrt{{\left(x_{i}-x_{j}\right)}^{2}+{\left(y_{i}-y_{j}\right)}^{2}+{\left(z_{i}-z_{j}\right)}^{2}}$$

where d denotes the distance between the COM of the i-th nitrogen molecule and the j-th solid framework atom (encompassing Si, O, or C atoms). Based on the effective range of van der Waals interactions (corresponding to the first coordination shell of the solid–gas interface), an adsorption cutoff distance of 0.5 nm was employed in this study. Consequently, if the calculated minimum relative distance satisfies d<0.5 nm, the nitrogen molecule is rigorously classified as being in the physically adsorbed state; conversely, it is determined to be in the free state.

## Figures and Tables

**Table 1:** Simulation cases of silica aerogel with different CNT doping fractions.

CNTs Fraction	5%	10%	15%	20%
Simulation model	** 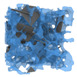 **	** 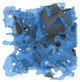 **	** 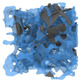 **	** 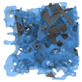 **
Number of C atoms	6300	12,600	18,900	25,200

**Table 2:** Interaction parameters [8].

Pair	ε/meV	σ/Å	q/e
Si-Si	1.73	4.05	0.6
O-O	9.88	2.86	−0.3
C-C	2.84	3.40	0

## Data Availability

Data is provided within the manuscript.
